# The miRNA world of polyomaviruses

**DOI:** 10.1186/1743-422X-10-268

**Published:** 2013-08-28

**Authors:** Ole Lagatie, Luc Tritsmans, Lieven J Stuyver

**Affiliations:** 1 Janssen Diagnostics, Turnhoutseweg 30, Beerse 2340, Belgium; 2 Janssen Research and Development, Turnhoutseweg 30, Beerse 2340, Belgium

**Keywords:** Polyomaviruses, microRNAs, Virus-host interaction, Immune evasion

## Abstract

Polyomaviruses are a family of non-enveloped DNA viruses infecting several species, including humans, primates, birds, rodents, bats, horse, cattle, raccoon and sea lion. They typically cause asymptomatic infection and establish latency but can be reactivated under certain conditions causing severe diseases. MicroRNAs (miRNAs) are small non-coding RNAs that play important roles in several cellular processes by binding to and inhibiting the translation of specific mRNA transcripts. In this review, we summarize the current knowledge of microRNAs involved in polyomavirus infection. We review in detail the different viral miRNAs that have been discovered and the role they play in controlling both host and viral protein expression. We also give an overview of the current understanding on how host miRNAs may function in controlling polyomavirus replication, immune evasion and pathogenesis.

## Review

### General overview of polyomaviruses

Polyomaviruses comprise a family of DNA tumor viruses. They are non-enveloped and have a circular, double stranded DNA genome of around 5,100 bp [1]. The virion consists of 72 pentamers of the capsid protein VP1 with a single copy of VP2 and VP3 associated to each pentamer [2,3]. Although originally categorized together with the *Papillomaviridae* under the designation of *Papovaviridae*, they were separated in 2000 by the International Committee on Taxonomy of Viruses to become two distinct families [4,5]. The first polyomavirus family member, murine polyomavirus (MuPyV), was discovered as a tumor agent in mice already in 1958, shortly followed by the first primate polyomavirus, Simian Virus 40 (SV40), which was discovered in 1960 [6,7]. Since the discovery of the first two human polyomaviruses, JC Virus (JCPyV) and BK Virus (BKPyV) in 1971, several new members of the polyomavirus family have been identified [8,9]. To date, complete genome reference sequences for 46 polyomaviruses have been deposited at Genbank. Of those, 12 are human polyomaviruses [10-13]. Several polyomaviruses have been associated to specific diseases, such as Progressive Multifocal Leukoencephalopathy (PML) for JCPyV, polyomavirus-associated nephropathy (PVAN) for BKPyV, Merkel cell carcinoma (MCC) for Merkel Cell Virus (MCPyV) and trichodysplasia spinulosa for Trichodysplasia spinulosa-associated Polyomavirus (TSPyV) [4,10,11,14-20]. One of the most striking observations is the fact that asymptomatic infection occurs during childhood which is followed ordinarily by life-long asymptomatic persistence [21]. It remains however puzzling how this latency state is changed into a reactivated state upon changes in the host immune system in some individuals.

**Figure 1:**
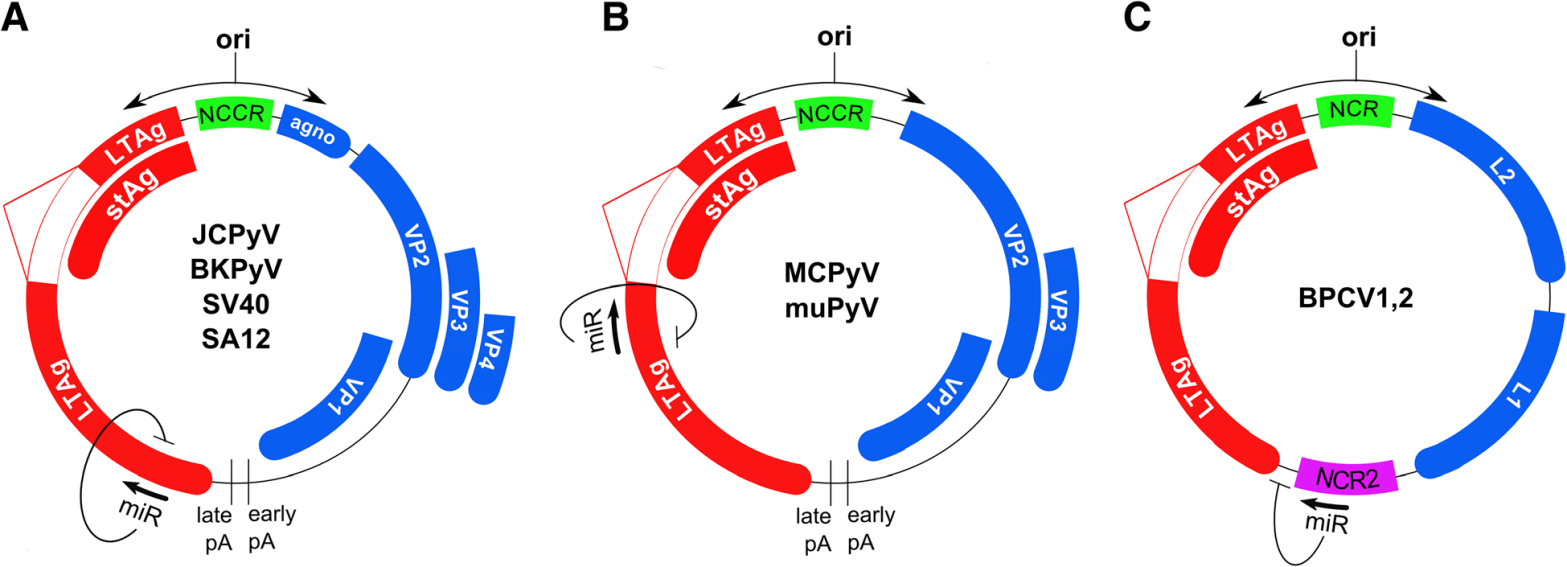
**Polyomavirus encoded miRNAs.** Different genomic locations of the polyomavirus encoded miRNAs have been described, but all of them are targeting the early transcript encoding the Large T-antigen (LTAg) and small T-antigen (stAg). Minor splicing variants of LTAg and MTAg (in MuPyV) are not presented. **A**, The alpha polyomaviruses JCPyV, BKPyV, SV40 and SA12 encode a miRNA located at the 3′ end of, and antisense to LT. Remark VP4 is included for completeness but has so far only been detected in SV40. **B**, MCPyV and MuPyV encode a miRNA located at the 5′ end of, and antisense to LT. **C**, BPCV, a virus that shares distinct characteristics of both the *Polyomaviridae* and the *Papillomaviridae* encodes a miRNA located in the second non-coding region (NCR2, indicated in pink) between the 3′ ends of the T-antigens and L1/L2.

All polyomaviruses have a similar genomic organization where the genome is almost evenly divided into an early and a late region encoded on opposite strands (Figure 1). In-between these two regions, a non-coding control region (NCCR) is present. This region encodes the origin of replication and contains the promoter elements that control transcription of both the early and late transcripts. The early region is transcribed soon after initial infection of the host cell and encodes at least the two proteins large T (tumor) antigen (LTAg) and small t antigen (stAg), which share the amino-terminal 75–80 amino acids. This shared part is encoded by exon 1 of the LTAg gene. Alternative splicing of the early messenger RNA (mRNA) transcript can result in up to three additional T antigens. In the case of JCPyV, three additional early proteins, T’135, T’136 and T’165, have been identified [22]. Also in BKPyV, SV40 and MCPyV, additional T antigens resulting from differently spliced early transcripts have been described [23-25]. LTAg exerts a pivotal function in viral replication and several domains can be identified in the protein that play specific roles in viral DNA replication and cell cycle control [26-30]. stAg also contains specific domains that bind to cellular proteins involved in cell cycle regulation, of which protein phosphatase 2A (PP2A) is best described [31,32]. The late region, as the name would suggest, is expressed later in the viral life cycle and results in the production of three capsid proteins VP1, VP2 and VP3, which will form the viral capsid. All of these proteins originate from the same mRNA transcript but are produced upon differential splicing and internal translation and use the same reading frame [33-35]. VP4, which also originates from the late transcript, has so far only been detected in SV40 where it functions as viroporin, promoting release of the virus from the cell [36,37]. The human polyomaviruses JCPyV and BKPyV and the monkey polyomaviruses SV40 and SA12 (and most probably other SV40-like viruses) also encode the agnoprotein on the leader region of the late transcript [38,39]. While no other polyomaviruses are known to encode this agnoprotein, murine and hamster polyomavirus encode a middle T antigen which functions as transforming protein [40]. Recently, also MCPyV was found to encode a protein phylogenetically related to this middle T antigen, called ALTO [41].

### Biogenesis and function of microRNAs

MicroRNAs (miRNAs) are RNAs of 20–23-nucleotide (nt) length that play a key role in several cellular processes. These non-coding RNAs typically silence gene expression by directing repressive protein complexes to the 3′ untranslated region (3′UTR) of target mRNA transcripts. Although originally discovered in the nematode *C. elegans*, they have been found to be expressed in several organisms, such as insects, nematodes, plants, humans and viruses [42-46]. Of particular interest is the role these small RNAs play in regulation of the innate immune response, adaptive immune cell differentiation, metabolism, apoptosis, cell proliferation, cancer and maintenance of homeostasis during stress [43,47].

MiRNAs are derived from longer precursor primary transcripts (pri-miRNAs) that are typically transcribed by RNA polymerase II (Pol II), which also is responsible for transcription of mRNAs. These pri-miRNAs contain at least one imperfect stem-loop hairpin structure and this hairpin structure is processed in the nucleus via the RNAse III-like endonuclease Drosha [48]. The newly formed ~60 nt hairpin, called pre-miRNA is exported from the nucleus into the cytosol via the RAN-GTPase Exportin-5 [49,50]. In the cytoplasm, this pre-miRNA is recognized and cleaved by the RNAse III-like endonuclease Dicer resulting in an RNA duplex, typically having short (~2nt) 3′ overhangs [51,52]. One of the two strands of this ~22nt duplex RNA, called the miRNA or “guide” strand is loaded into the multiprotein RNA-induced silencing complex (RISC). The other strand, called the “star” (*) or “passenger” strand is energetically less favored to enter RISC and is therefore typically found at lower steady state levels. A key component of RISC is the Argonaute (Ago) protein, which associates with the guide strand, thereby directing the complex to the target sequence through Watson-Crick base pairing [51-53]. MiRNA binding sites are usually located in the 3′ UTR and are often present in multiple copies. Most animal miRNAs bind imperfectly with the target mRNA, although a key feature of recognition involves base-pairing of miRNA nucleotides 2–8, representing the seed region [54]. The degree of miRNA-mRNA complementarity is a key determinant of the further process. Perfect or near perfect complementarity may lead to Ago-catalyzed cleavage of the mRNA strand, whereas imperfect complementarity leads to translational repression, which is thought to be the default mechanism by which miRNAs repress gene expression [54-56]. Although multiple Argonaute proteins are present in mammals, only Ago2 is shown to have mRNA cleavage activity [57].

### Polyomaviruses encoded miRNAs

**Figure 2:**
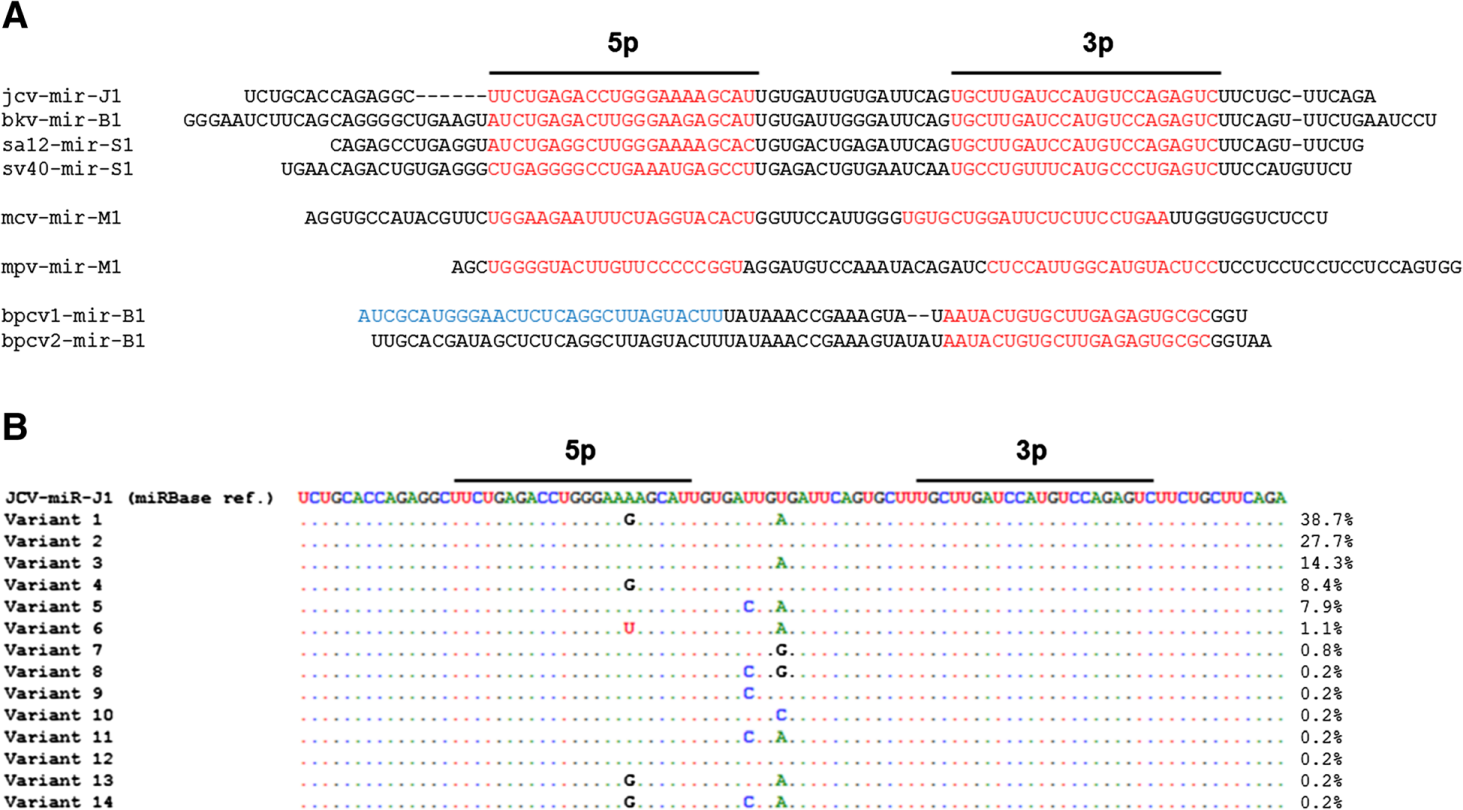
**Sequences of polyomavirus miRNAs. A**, Sequence comparison of the different polyomavirus miRNAs. The mature 5p and 3p miRNAs are indicated in red. Remark that for Bpcv1 and Bpcv2 also a 5p miRNA was observed, but no mapping of the sequence was performed. The sequence that was used for probing of this 5p miRNA is indicated in blue. **B**, Sequence variants observed in JCPyV. A total of 643 JCPyV nucleotide sequences were retrieved from NCBI and aligned using ClustalW algorithm in BioEdit. Relative abundance (%) of each sequence variant was calculated. JCV-miR-J1 sequence from miRBase V20 was used as reference sequence.

Given the role miRNAs play in several cellular processes, it was perhaps not surprising that viruses would employ them to modulate both their own gene expression and that of their host cells [58]. Since the discovery of the first viral encoded miRNAs in Epstein-Barr virus (EBV), 493 viral miRNAs have been identified and entered in miRBase (http://www.mirbase.org), most of them encoded by DNA viruses which replicate in the nucleus, such as herpesviruses and polyomaviruses [59-61]. While herpesviruses encode between 0 (Varicella Zoster Virus) and 68 (Rhesus lymphocryptovirus) miRNAs, all polyomaviruses where miRNAs have been investigated encode only 2 mature miRNAs, originating from one pre-miRNA (according to miRBase version 20) (Table 1, Figure 1 and Figure 2A).

**Table 1:** microRNAs in polyomaviruses

**Virus**	**miRNAs**	**miRBase V20 Accession No.**	**Genomic location (nt position in reference sequence)**	**mRNA targets**	**References**
JCPyV	jcv-miR-J1-5p	MIMAT0009147	3′ end LTAg (2682–2767 in NC_001699.1)	LTAg, stAg^2^ ULBP3	[63,65,66]
	jcv-miR-J1-3p	MIMAT0009148			
BKPyV	bkv-miR-B1-5p	MIMAT0009149	3′ end LTAg (2808–2909 in NC_001538.1)	LTAg, stAg^2^	[63,65,66,78]
	bkv-miR-B1-3p	MIMAT0009150			
SV40	sv40-miR-S1-5p	MIMAT0003344	3′ end LTAg (2776–2863 in NC_001669.1)	LT, stAg DMWD^2^, C20orf27^2^	[64,85]
	sv40-miR-S1-3p	MIMAT0003345			
SA12	sa12-miR-S1-5p	n.a.	3′ end LTAg (2786–2869 in NC_007611.1)	LTAg^2^, stAg^2^	[62]
	sa12-miR-S1-3p				
MCPyV	mcv-miR-M1-5p	MIMAT0010150	5′ end LTAg (1168–1251 in JN383838.1)	LTAg^1^ AMBRA2^2^, RBM9^2^, MECP2^2^, PIK3CD^2^, PSME^2^3 and RUNX2^2^	[68,69]
	mcv-miR-M1-3p	MIMAT0010151			
MuPyV	mpv-mir-M1-5p	n.a.	5′ end LTAg (1137–1269 in NC_001515.1)	LTAg, MTAg, stAg	[67]
	mpv-mir-M1-3p				
BPCV1	Bpcv-mir-B1-3p	MIMAT0020276	between the 3′ ends of the T-antigens and L1/L2 (4917–4987 in NC_010107.1)	T-antigens^1^	[72]
BPCV2	Bpcv-mir-B2-3p	MIMAT0020277	between the 3′ ends of the T-antigens and L1/L2 (no reference available)	T-antigens^1^	[72]

^1^ experimental evidence using reporter constructs only.

^2^ predicted target, no experimental evidence.

n.a. not available.

The closely related polyomaviruses JCPyV, BKPyV, SV40, and SA12 have been shown to encode a single pre-miRNA that maps to the late strand of the viral genome and is found downstream of the late polyadenylation (pA) site (Figure 1A) [62-66]. The mature miRNAs are located at the 3′end of the second LTAg exon, which is transcribed in the viral early transcript and the miRNAs are therefore completely complementary to the early mRNA. As would be predicted based on this property, it was shown for SV40 that the miRNAs direct cleavage of this early mRNA resulting in reduced protein expression of LTAg and stAg [64]. Although there is very high sequence similarity among the miRNAs encoded by JCPyV, BKPyV and SA12, the 5′ and 3′ mature SV40 miRNAs only have 50 and 77% identity to the JCPyV and BKPyV sequences, respectively [63] (Figure 2A). In contrast the 3′ miRNA is 100% conserved among JCPyV, BKPyV and SA12. Consequently, in case of co-infection of e.g. JCPyV and BKPyV no differentiation of miRNA activity can be made. Remarkably, for all these miRNAs both the 5p and 3p arms are generated from the pre-miRNA hairpin and large amounts of pre-miRNA are accumulating in the cell, indicating inefficient processing of this pre-miRNA [58].

Up till now, limited knowledge is available on miRNAs in other polyomavirus. In murine polyomavirus (MuPyV) and Merkel Cell Virus (MCPyV), no sequence homology was found with the miRNAs identified in the alpha polyomaviruses. However, in both viruses, a miRNA was identified that is located complementary to the early mRNA transcript but located more upstream, at the 5′ end of the second LTAg exon (Figure 1B). As was the case for the alpha polyomavirus encoded miRNAs, the MuPyV miRNA was also shown to direct cleavage of the early mRNA, consequently downregulating protein expression levels of LTAg, as well as MTAg and stAg [67]. For MCPyV targeting of the early mRNA transcripts by its miRNA was demonstrated using reporter constructs [68,69]. Based on these observations, it appears that, despite differences in sequence or genomic location, polyomavirus miRNAs all share the property that they target the early mRNA transcript.

Recently, a miRNA has also been identified in Bandicoot Papillomatosis Carcinomatosis Virus (BPCV), a virus that shares distinct characteristics of both the *Polyomaviridae* and the *Papillomaviridae*. BPCV is a marsupial virus associated with papillomas and carcinomas in western barred bandicoots (*Perameles bougainville*) [70,71]. This miRNA is not located in and complementary to the early transcript. Instead, it is located within the second non-coding region between the 3′ ends of the T-antigens and L1/L2 (Figure 1C). Furthermore, this BPCV miRNA was shown to have its own promoter located within ~60 nt of the base of the stem portion of the predicted hairpin pre-miRNA. Despite the different genomic organization, this miRNA was also shown to downregulate BPCV T-Antigens through targeting of the 3′UTR of the BPCV early transcript [72].

In order to get a snapshot of sequence variation in the miRNA region, an analysis was performed on a set of 643 publicly available nucleotide sequences of JCPyV isolates (Figure 2B). Besides some minor variants that only were observed very rarely, 3 variants were identified that appeared to be widespread. Two of these polymorphisms are located in the loop region between the 5′ and 3′ miRNAs and one is located in the 5′ miRNA. Of particular interest is the polymorphism in the 5′ miRNA as this appears to represent one of the 3 differences between the 5′ miRNAs of JCPyV and BKPyV, indicating that these miRNAs are even more similar than originally being described [63]. Whether these polymorphisms impact the stability of the pre-miRNA or the functionality of the mature miRNAs needs to be investigated. For SV40, a recent study has demonstrated that different miRNA variants exist that have different host target repertoires, while their autoregulatory activity on virus-encoded early gene products is preserved [73].

Time-course expression analysis of the polyomavirus miRNAs all show miRNA expression late in infection [63,64,67]. Together with the fact that the miRNAs are encoded on the late strand, it was suggested that the pre-miRNA emanates from the viral late pre-mRNA [58,64]. Conversely, the late polyadenylation site of polyomaviruses is located between the 3′ ends of VP1 and LTAg, suggesting that the more downstream encoded miRNA is not transcribed on the late mRNA transcript. It was however shown that the late polyadenylation site exerts only very weak polyadenylation efficiency specifically at late times in infection, thereby allowing long primary transcripts to be produced [74-76]. Furthermore, production of these long transcripts appears to lower the accumulation of early mRNAs [77]. Whether polyomavirus miRNA expression is dependent on this late-strand read-through or whether it is produced from a so far unknown independent transcript, remains to be determined.

### Autoregulatory role of Polyomavirus miRNAs

The fact that different polyomaviruses encode miRNAs that may differ in sequence and their genomic location, but all target their respective early mRNA transcript indicates an important role of these small regulatory RNA fragments in the life cycle and possibly also host interaction of this virus family. It was shown that expression of the miRNAs reduces expression of LTAg [63,64,67,69]. As LTAg plays an important role in recognition of infected cells by the cytotoxic T lymphocytes (CTL), it was originally thought that miRNA mediated downregulation of the LTAg mainly plays a role in lowering recognition by the immune system [64]. However, no differences in infection could be observed in specifically designed SV40 or MuPyV miRNA mutants, consequently questioning the importance of these miRNAs [64,67]. Recently, however, the importance of miRNA mediated downregulation of LTAg was re-established as it was shown that downregulation of BKPyV LTAg by its own miRNA is one of the main factors controlling viral replication [78]. Reduction of the miRNA expression level through rearrangements in the NCCR was shown to increase viral replication and as such expression of the viral miRNA might be a crucial element in establishing or maintaining viral latency or persistence [78]. This high degree of regulation is rather atypical for miRNAs as they usually exert a more fine-tuning role. This might be attributed to the fact that these miRNAs have full complementarity to their target, thereby promoting degradation of the mRNA strand, rather than the more common translational inhibition upon imperfect complementarity. It will be interesting to see whether this essential role of the viral miRNA in controlling the viral replication is a common theme among all polyomaviruses.

### Regulation of host factors by Polyomavirus miRNAs

**Figure 3:**
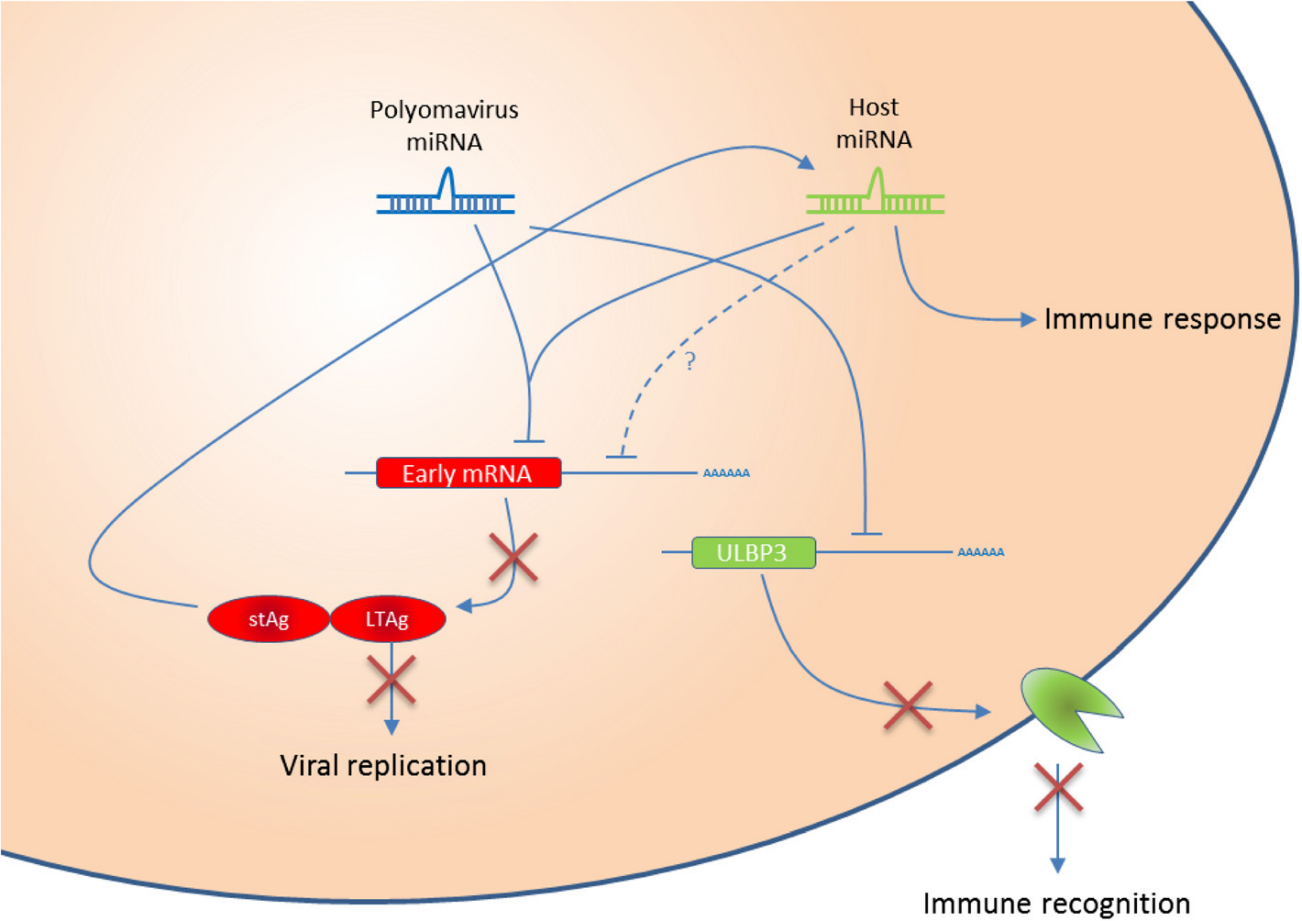
**Overview of viral and host miRNA functions related to Polyomavirus infection.** Polyomavirus encoded miRNAs exert an autoregulatory role through targeting of LTAg, which plays an essential role in viral replication. This viral miRNA also plays a role in regulating the host immune response by targeting host factors, such as the stress-induced ligand ULBP3, that are essential for recognition of infected cells by the immune system. Host miRNAs can mimic the polyomavirus miRNA, thereby also influencing the expression of LTAg and consequently viral replication, but they might also affect viral protein synthesis by targeting the viral transcripts through binding at the 3′UTR of the viral transcripts. Next to this virus specific role, miRNAs are also shown to play a more general role in the immune response upon viral infection.

Next to the autoregulatory role of the viral miRNAs, it was also suggested that viral miRNAs could play an important role in controlling specific host factors, possibly resulting in immune evasion and viral persistence [79]. The 3′ miRNA of JCPyV (and BKPyV as they are identical) specifically downregulates the expression of ULBP3 (UL16-binding protein 3) through binding to its 3′UTR, resulting in reduced translation of the transcript (Figure 3) [65,66]. ULBP3 is one of the stress-induced ligands that are recognized by the powerful killer receptor NKG2D (natural killer group 2, member D), which is expressed by NK cells and CD8^+^ T cells, resulting in target cell killing [80-82]. It was demonstrated that JCV-miR-J1-3p mediated downregulation of ULBP3 leads to escape from NKG2D-mediated killing by NK cells [65,66]. Remarkably, it was also shown that the miRNAs derived from the herpes viruses human cytomegalovirus (HCMV), Kaposi's sarcoma-associated herpesvirus (KSHV) and EBV target MICB (MHC Class I chain-related protein B), another NKG2D ligand [83,84]. Next to this targeting of NKG2D ligands, it was shown more recently that SV40-miR-S1-5p might negatively regulate the expression of host proteins DMWD and C20orf27 through targeting of their respective 3′ UTR [85]. As this was demonstrated using luciferase reporter assays only, determination of actual protein levels will be required to confirm this interaction. The functional relevance of this miRNA dependent host factor control also remains to be elucidated as little is known of both proteins, except for a role of DMWD in myotonic dystrophy [86]. For MCPyV potential host targets have been identified through bio-informatic approaches, but so far no experimental evidence has been obtained to demonstrate targeting of host mRNAs by the MCPyV miRNA or to demonstrate a role for any of these putative target genes in the viral life cycle. Among these predicted targets are AMBRA1, RBM9, MECP2, PIK3CD, PSME3 and RUNX1 (Table 1) [68].

### Polyomaviruses influence host miRNAs

One of the mechanisms viruses use to disturb the physiological functions of host cells is by altering the levels of host miRNAs. Since miRNAs have been shown to function as oncogenes or tumor suppressors, this mechanism might be of particular interest for tumor-inducing polyomaviruses, such as SV40 and MCPyV [87]. Indeed, it was shown that expression of SV40 stAg results in an induction of the human hsa-miR-27a in a PP2A dependent way, with hsa-miR-27a being a potent tumor promoter involved in cell proliferation [88]. Similar examples of virally controlled host miRNA expression exists for the human papillomavirus HPV-31 where the virus appeared to downregulate the expression of hsa-miR-145, which in turn results in increased viral genome amplification [89]. A more general control of host miRNA expression by polyomavirus LTAg might also not be excluded as this viral protein is known to affect RNA polymerase II-dependent transcription, which is required for production of pri-miRNAs [48,90]. It will be of particular interest to see whether miRNA profiling studies where infected and non-infected cells are compared, reveal new host miRNAs affected by a specific polyomavirus. Similar studies with other viruses have demonstrated that this might be a successful approach to identify new host miRNAs involved in viral infection [91-94].

### Host miRNAs influence polyomaviruses

Although still a rather unexplored domain in the polyomavirus field, work on other viruses has shown that not only the virus autoregulates viral mRNAs or regulates cellular mRNAs or miRNAs, but that also host miRNAs play an important role in the regulation of the expression of specific viral gene products (Figure 3). This might also play a role during viral latency or tumorigenesis. One mechanism the host might employ is the expression of functional orthologs of the viral miRNA. For SV40 it was shown that hsa-miR-423-5p may act as a functional ortholog of SV40-miR-S1-5p, which shares identical seed sequence [85]. Together with the fact that the viral miRNA downregulates LTAg, this would then imply that hsa-miR-423-5p negatively regulates this antigenic protein, thereby reducing immune response against the virus and limiting the viral replication rate [64]. Whereas SV40 is the only polyomavirus so far for which this has been demonstrated, the existence of these functional orthologs has also been described for the Human Immunodeficiency Virus-1 (HIV-1) [85], the Herpesviruses Kaposi’s sarcoma-associated herpesvirus and Marek’s Disease Virus [95-98], and Epstein-Barr virus [99]. Although the existence of these functional orthologs appears to be a more general phenomenon, it is not clear whether these host miRNAs really evolved as a mechanism to combat specific viral infections or whether the virus – which likely evolves faster – in fact evolved to mimic specific host miRNAs.

Although the host can affect the viral life cycle through miRNAs that mimic the viral miRNAs, the host cell might also express miRNAs that specifically recognize sequences in the viral genome. This mechanism is of particular interest for disease related viruses as this cellular miRNA might be a promising drug target. The latter has been demonstrated already for hsa-miR-122 in the context of Hepatitis C Virus (HCV) infection [100,101]. This host miRNA is highly abundant in the liver and appears to bind specific sites in the HCV genome, thereby protecting it from nucleolytic degradation [102]. Administration of locked nucleic acid-modified antisense oligonucleotides resulted in effective viral suppression *in vivo*[100,103]. So far, no miRNAs have been identified that target specific polyomavirus sequences.

Recent work has also identified hsa-miR-155 as an important host cell miRNA involved in a more general role in the immune response upon viral infection. Two reports have shown independently that this miRNA is essential for CD8^+^ T cell responses upon infection with lymphocytic choriomeningitis virus (LCMV), where it appeared to influence T cell survival upon viral infection [104,105]. Whether hsa-miR-155 also plays a role in the immune control of polyomaviruses and more specifically in polyomavirus-induced diseases that are dependent on changes in the host immune system is of particular interest but remains to be determined.

## Conclusions

Although miRNAs have only been discovered 20 years ago, they have been recognized as important regulators of several cellular processes. We have provided an overview of what is known so far on the role miRNAs play in the biology of polyomaviruses. Virally encoded miRNAs have been described in several polyomavirus and despite the fact that different locations on the genome have been discovered, they all appear to target the early mRNA transcript encoding the T-antigens. This conserved functionality already indicated an important role for these miRNAs but only recently it was discovered how this relates to control of viral replication. Next to this autoregulatory role, the polyomavirus miRNAs have been shown to target host factors as well, thereby possibly modulating the host response. It will be interesting for future work to better characterize these regulatory mechanisms, also in other polyomaviruses, as well as to study the role these play in polyomavirus related diseases. The role of host miRNAs in polyomavirus infection has only been studied to a very small extent so far. As was already the case for other viruses, it is likely that potential antiviral drug targets can be found among miRNAs, emphasizing the therapeutic potential of anti-miRs in polyomavirus related diseases. Taken together, miRNAs are shown to be essential factors in the control of polyomavirus replication and the interaction with their host. Whereas the foundation in understanding the role of miRNAs in polyomavirus biology has been laid now, this remains a rather unexplored domain with lots of potential for future research.

## Abbreviations

MuPyV: Murine polyomavirus; SV40: Simian virus 40; JCPyV: JC Virus; BKPyV: BK Virus; PML: Progressive multifocal leukoencephalopathy; PVAN: Polyomavirus-associated nephropathy; MCC: Merkel cell carcinoma; MCPyV: Merkel cell virus; TSPyV: Trichodysplasia spinulosa-associated Polyomavirus; NCCR: Non-coding control region; LTAg: Large T (tumor) antigen; stAg: Small T antigen; miRNA: microRNA; 3′UTR: 3′untranslated region; RISC: RNA-induced silencing complex; Ago: Argonaute; EBV: Epstein-Barr virus; CTL: Cytotoxic T lymphocytes; ULBP3: UL16-binding protein 3; NKG2D: Natural killer group 2, member D; NK: Natural killer; HCMV: Herpes viruses human cytomegalovirus; KSHV: Kaposi's sarcoma-associated herpesvirus; MICB: MHC class I chain-related protein B; BPCV: Bandicoot Papillomatosis Carcinomatosis virus; HIV-1: Human immunodificiency virus-1; HCV: Hepatitis C virus; LCMV: Lymphocytic choriomeningitis virus.

## Competing interests

Authors are current employees of Janssen Diagnostics BVBA, a Johnson and Johnson Company and may own stock or stock options in that company.

## Authors’ contributions

OL contributed to the literature research, analysis and interpretation of the literature data as well as writing of the final review. LT and LJS contributed to revising the manuscript critically for important intellectual content and gave final approval of the version. All authors read and approved the final manuscript.
